# Synergistic Effect of Work Function and Acoustic Impedance Mismatch for Improved Thermoelectric Performance in GeTe-WC Composite

**DOI:** 10.1021/acsami.2c11369

**Published:** 2022-09-21

**Authors:** Ashutosh Kumar, Preeti Bhumla, Artur Kosonowski, Karol Wolski, Szczepan Zapotoczny, Saswata Bhattacharya, Krzysztof T. Wojciechowski

**Affiliations:** † Lukasiewicz Research Network - Krakow Institute of Technology, Kraków 30-011, Poland; ‡ Faculty of Materials Science and Ceramics, AGH University of Science and Technology, Kraków 30-059, Poland; § Department of Physics, Indian Institute of Technology Delhi, New Delhi 110016, India; ∥ Faculty of Chemistry, Jagiellonian University, Gronostajowa 2, Kraków 30-387, Poland

**Keywords:** composite thermoelectrics, acoustic impedance mismatch, Kapitza radius, interface thermal resistance, work function, Kelvin probe force microscope, density functional theory

## Abstract

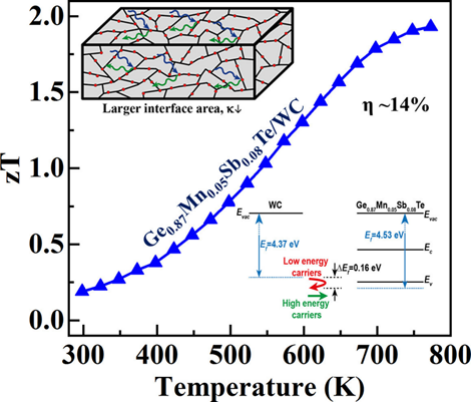

The preparation of composite materials is a promising methodology for concurrent optimization of electrical and thermal transport properties for improved thermoelectric (TE) performance. This study demonstrates how the acoustic impedance mismatch (AIM) and the work function of components decouple the TE parameters to achieve enhanced TE performance of the (1-*z*)Ge_0.87_Mn_0.05_Sb_0.08_Te-(*z*)WC composite. The simultaneous increase in the electrical conductivity (σ) and Seebeck coefficient (α) with WC (tungsten carbide) volume fraction (*z*) results in an enhanced power factor (α^2^σ) in the composite. The rise in σ is attributed to the creation of favorable current paths through the WC phase located between grains of Ge_0.87_Mn_0.05_Sb_0.08_Te, which leads to increased carrier mobility in the composite. Detailed analysis of the obtained electrical properties was performed via Kelvin probe force microscopy (work function measurement) and atomic force microscopy techniques (spatial current distribution map and current–voltage (*I–V*) characteristics), which are further supported by density functional theory (DFT) calculations. Furthermore, the difference in elastic properties (i.e., sound velocity) between Ge_0.87_Mn_0.05_Sb_0.08_Te and WC results in a high AIM, and hence, a large interface thermal resistance (*R*_int_) between the phases is achieved. The correlation between *R*_int_ and the Kapitza radius depicts a reduced phonon thermal conductivity (κ_ph_) of the composite, which is explained using the Bruggeman asymmetrical model. Moreover, the decrease in κ_ph_ is further validated by phonon dispersion calculations that indicate the decrease in phonon group velocity in the composite. The simultaneous effect of enhanced α^2^σ and reduced κ_ph_ results in a maximum figure of merit (*zT*) of 1.93 at 773 K for (1-*z*)Ge_0.87_Mn_0.05_Sb_0.08_Te-(*z*)WC composite for *z* = 0.010. It results in an average thermoelectric figure of merit (*zT*_av_) of 1.02 for a temperature difference (Δ*T*) of 473 K. This study shows promise to achieve higher *zT*_av_ across a wide range of composite materials.

## 1 Introduction

The majority of the energy generated from various energy sources is wasted in the form of so-called waste heat. Therefore, technology that can utilize it is essential for the efficient use and production of energy in general. Thermoelectric (TE) energy conversion technology that can harvest waste heat energy using temperature gradient without emitting pollution is a propitious solution for waste heat recovery and niche power resources.^1,2^ The usefulness of TE materials depends on their thermoelectric figure of merit (*zT*), defined as
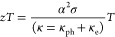
1where *T* is absolute temperature, α^2^σ is known as a power factor that includes Seebeck coefficient (α) and electrical conductivity (σ), and κ is total thermal conductivity consisting that can be defined as κ = κ_e_ + κ_ph_, where κ_e_ is an electronic thermal conductivity and κ_ph_ is phonon thermal conductivity. The strong coupling between σ, α, and κ_e_ creates a challenge to achieve a high *zT* in a pristine, unmodified material.^3^ The electrical properties have been optimized through several concepts regarding transport mechanisms of charge carriers to achieve enhanced α^2^σ in single-phase materials.^4−6^ Furthermore, the reduction in κ_ph_ has been presented in the literature through several strategies that amplify phonon scattering, that is, the introduction of lattice defects in the structure,^7,8^ creation of artificial superlattices,^9^ utilization of mass fluctuation/disorder effects,^10^ nanostructurization,^11^ or preparation of composite materials.^12,13^ However, such concepts for lowering κ_ph_ will also alter the charge transport and most likely reduce σ due to the scattering of carriers.^14^

Moreover, the preparation of composite materials is promising for simultaneous optimization of electrical and thermal transport properties to obtain an improved TE performance. This approach allows the utilization of a few transport phenomena that can result in improvement of the σ/κ ratio.^15−21^ The simultaneous filtering of the charge carrier and enhanced phonon scattering at the interface between the Bi_0.4_Sb_1.6_Te_3_-Cu_2_Se nanocomposite results in an enhanced *zT* (∼1.6 at 488 K).^16^ Kim et al. reported an improved *zT* (∼1.85) in the PbTe-PbSe composite because of the synergistic effect of reduced κ_ph_ and enhanced α^2^σ.^22^ A notable reduction in κ_ph_ was also observed in several composites with nanostructured secondary phase and is mainly attributed to the quantum size effects.^16,23−27^ In composite materials, the interface thermal resistance (*R*_int_), which originates from the acoustic impedance mismatch (AIM) between the phases is rarely considered for the optimization of κ_ph_.^28^ However, thermal resistance at the phase boundary is often used in the description of the heat transport in ceramic and polymer composites, including ZnS/diamond,^28^ SiC/Al^29^, and glass/epoxy.^30^ These reports along with our previous studies on composite materials demonstrate that interface thermal resistance between the phases of the composite (and *R*_int_ as a parameter) is crucial in designing TE composite materials with the desired κ_ph_.^12^

GeTe-based materials are promising for TE application in the mid-temperature range (500–800 K). However, pristine GeTe suffers from the intrinsic Ge vacancies that result in a high hole carrier concentration (∼10^21^ cm^–3^), high thermal conductivity (∼8 W·m^–1^·K^–1^), and low Seebeck coefficient (∼30 μV·K^–1^) and hence poor *zT*.^31^ Several innovative approaches have been demonstrated in recent times to achieve enhanced thermoelectric performance in GeTe including manipulation of Ge vacancies,^32^ band convergence,^33−35^ crystal structure modification,^36,37^ resonance-level doping^38,39^ high-entropy concept,^40^ and so forth and are based on the atomic doping strategies. Herein, we demonstrate a novel composite approach that considers the optimized GeTe (Ge_0.87_Mn_0.05_Sb_0.08_Te via band-structure and lattice dynamics engineering^41^) as matrix and tungsten carbide (WC: possesses higher electrical and thermal conductivity than Ge_0.87_Mn_0.05_Sb_0.08_Te) as the second phase. In composite, interface between phases plays a vital role in determining the electrical and thermal conductivity. However, the role of interface has been neglected or underestimated in optimizing the performance of a thermoelectric material. The present study focuses on the effect of WC addition on thermoelectric properties of the Ge_0.87_Mn_0.05_Sb_0.08_Te-WC composite considering the role of workfunction and interface thermal resistance on electrical and thermal conductivity in the Ge_0.87_Mn_0.05_Sb_0.08_Te-WC composite. The effect of WC on electrical transport has been analyzed using the Kelvin probe and atomic force microscope via measuring the work function of both materials, current–voltage characteristics, and current distribution map. Furthermore, the electronic band structure for the Mn-Sb co-doped GeTe-WC composite is calculated using the density functional theory (DFT). The large difference in elastic properties between Ge_0.87_Mn_0.05_Sb_0.08_Te and WC has been used to control κ_ph_ in the composite using the interface thermal resistance (*R*_int_) between the phases, estimated from the acoustic impedance model (AIM) and the Debye model. The κ_ph_ in the composite is further analyzed using the Bruggeman asymmetrical model, which considers *R*_int_ between the phases. Furthermore, the phonon dispersion calculation for the Mn-Sb doped GeTe-WC composite is also performed to establish the decrease in κ_ph_.

## 2 Results and Discussion

### 2.1 Structural Characterization

**Figure 1:**
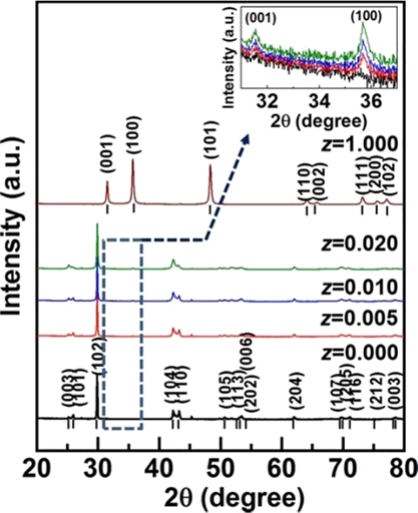
X-ray diffraction pattern for the (1-*z*)Ge_0.87_Mn_0.05_Sb_0.08_Te-(*z*)WC composite. Inset shows the zoom-in image in the log-scale depicting the presence of WC. The Miller indices and Bragg’s position for Ge_0.87_Mn_0.05_Sb_0.08_Te and WC are marked.

X-ray diffraction pattern of the (1-*z*)Ge_0.87_Mn_0.05_Sb_0.08_Te-(*z*)WC composite is shown in Figure 1. Ge_0.87_Mn_0.05_Sb_0.08_Te has a rhombohedral structure (space group: *R3m*) at 300 K, with the lattice parameters *a* = *b* = 4.1709 Å, *c* = 10.5612 Å in a hexagonal configuration. The reflection intensity of WC is not prominently observed because of its low volume fraction in the composite. However, the main reflections (001) and (100) of WC are revealed in the log-scale (inset of Figure 1) and confirm its presence in the composite. It is seen that the reflections corresponding to the WC phase enlarge with the increase in the WC volume fraction (*z*) in the composite.

**Figure 2:**
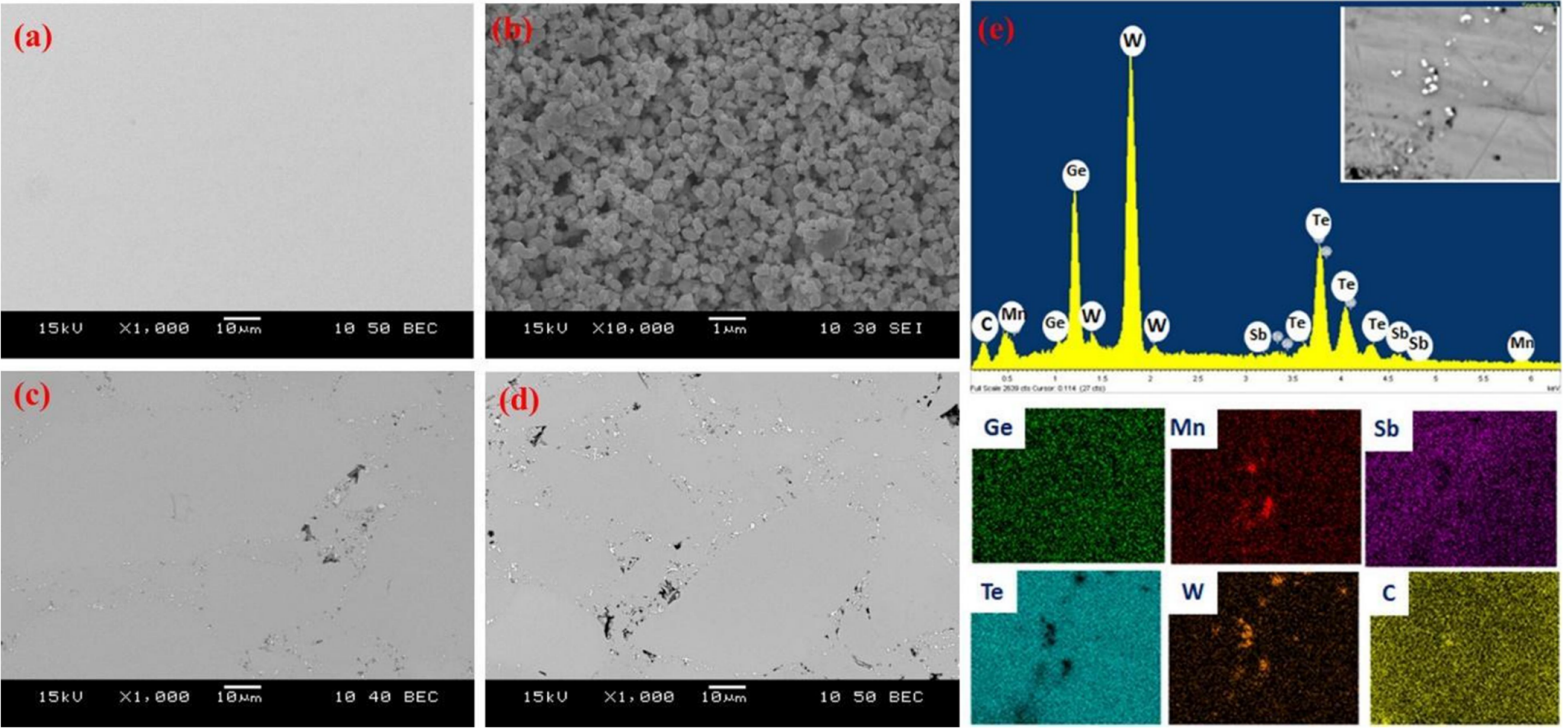
SEM images for (a) Ge_0.87_Mn_0.05_Sb_0.08_Te polished pellet (b) WC powder, and (1-*z*)Ge_0.87_Mn_0.05_Sb_0.08_Te-(*z*)WC composite with (c) *z* = 0.010, (d) *z* = 0.020 and (e) EDS spectra for *z* = 0.010 is shown. Inset shows the zoom-in image for *z* = 0.010. Corresponding elemental mapping for each element is also shown.

Figure 2a–d shows the scanning electron microscopy (SEM) image for the polished surface of the

(1-*z*)Ge_0.87_Mn_0.05_Sb_0.08_Te-(*z*)WC composite. The sintered Ge_0.87_Mn_0.05_Sb_0.08_Te is homogenous and one does not observe pores and impurity phases, as well as grain boundaries in nonchemically etched samples Figure 2a. The microstructure of WC powder used for preparing the composites is shown in Figure 2b. It is observed that WC particles are uniformly shaped with size in the range of 150–200 nm. The WC particles segregate uniformly at the grain boundary of Ge_0.87_Mn_0.05_Sb_0.08_Te (Figure 2c, d), making them visible. Their amount increases with the higher WC volume fraction (*z*). One can also observe irregular pores at points of contact of three or more grains. However, the pores are rarely observed at grain boundaries between two adjacent grains. Therefore, the samples’ relative density is very high and lies in the range of 98–99%, and the pores should not significantly determine transport properties. Figure 2e shows the energy dispersive X-ray spectra (EDS) of the composite sample with *z* = 0.010. The zoom-in image for the same sample is shown in the inset of Figure 2e. All elements within the Ge_0.87_Mn_0.05_Sb_0.08_Te-WC sample are confirmed by the EDS analysis, as shown in Figure 2e. Other elements were not detected. One cannot also observe the presence of dispersed W and C in the Ge_0.87_Mn_0.05_Sb_0.08_Te grains. The structural and microstructural analysis shows the existence of both individual phases in the composite samples. It further confirms the satisfying purity of the composite constituent and the high density of sintered materials.

### 2.2 Atomic Force Microscopy Analysis

**Figure 3:**
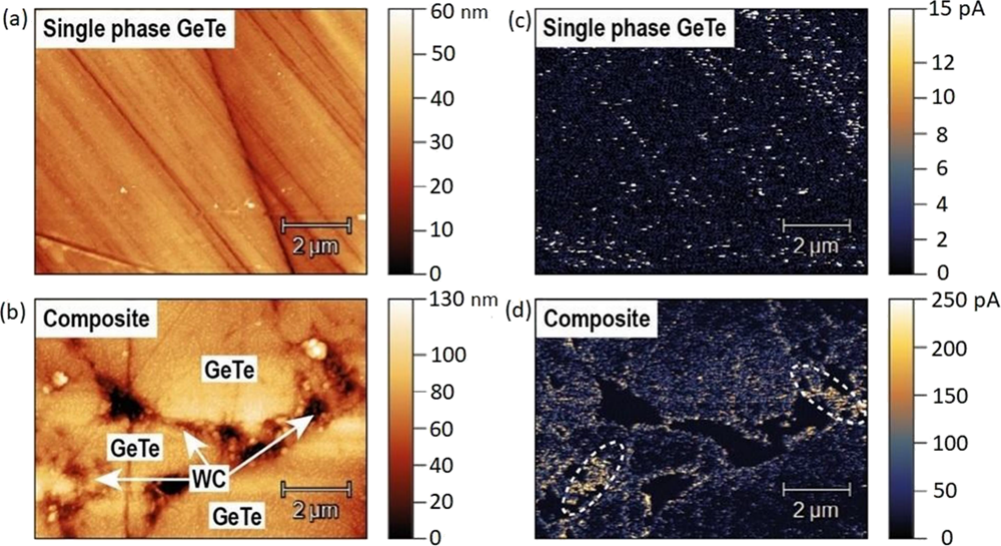
Surface topology (a, b) and the corresponding current distribution map (c, d) for single-phase Ge_0.87_Mn_0.05_Sb_0.08_Te and (1-*z*)Ge_0.87_Mn_0.05_Sb_0.08_Te-(*z*)WC composites with *z* = 0.02. GeTe abbreviations in the pictures designate the Ge_0.87_Mn_0.05_Sb_0.08_Te phase. Images were captured at 20 mV DC voltage.

Atomic force microscopy (AFM) analysis reveals more details concerning the microstructure and charge transport phenomena near the grain boundaries. The surface topology and corresponding current distribution for GeTe (Ge_0.87_Mn_0.05_Sb_0.08_Te) and GeTe-WC composites are shown in Figure 3. The analysis confirms that the pure (*z* = 0) polycrystalline Ge_0.87_Mn_0.05_Sb_0.08_Te phase (having GeTe structure) is homogenous and fine grains are well sintered, ensuring good electrical and thermal contacts (Figure 3a). On the other hand, it is seen that in the Ge_0.87_Mn_0.05_Sb_0.08_Te-WC composite, submicron WC particles are located between GeTe grains at their boundaries (Figure 3c), as seen in SEM images. The WC particles are uniformly distributed and are well attached, confirming good adhesion to GeTe grains. Furthermore, the WC particles create larger aggregates in some places. There are observed voids (pores) at places of WC aggregates and sharp corners of the grains.

**Figure 4:**
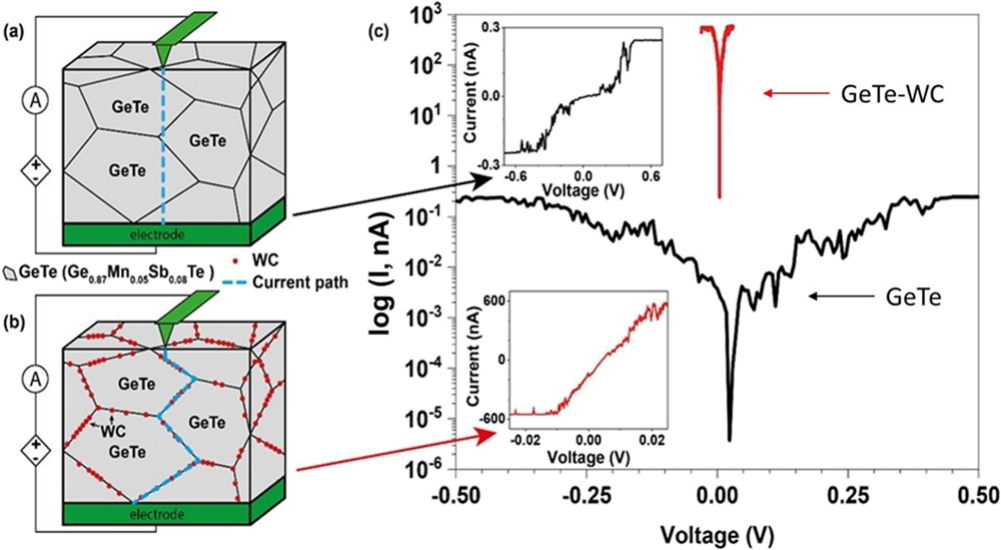
Scheme of the local microstructure for (a) single-phase GeTe and (b) composite with 2 vol % of WC. (c) Recorded *I–V* curves for GeTe and GeTe-WC composites (GeTe corresponds to the Ge_0.87_Mn_0.05_Sb_0.08_Te phase).

Single-phase Ge_0.87_Mn_0.05_Sb_0.08_Te as well as (1-*z*)Ge_0.87_Mn_0.05_Sb_0.08_Te-(*z*)WC with *z* = 0.02 composite was subjected to the additional analysis utilizing the AFM method to determine the current distribution on the surface of the material (Figure 3c, d) and to measure the current–voltage (*I–V*) characteristic (Figure 4). The current distribution map shows that the current in single-phase GeTe (Figure 3c) is distributed almost uniformly throughout the whole investigated area. However, for composite materials (Figure 3d), the current is significantly higher near the grain boundary of Ge_0.87_Mn_0.05_Sb_0.08_Te (marked with a dashed closed path) in comparison to within the grains. Nevertheless, there is still a noticeable amount of the current flowing through the volume of the Ge_0.87_Mn_0.05_Sb_0.08_Te. This indicates that the percolation threshold is not reached.

Figure 4a, b shows the out-of-plane measurement configuration used for both materials, where the current was flowing from the AFM tip through the polycrystalline material to the flat electrode at the opposite side of the sample. It is known that charge carriers follow a low resistance path. In GeTe (Figure 4a), the possible low resistance path is represented via a straight line. However, in the GeTe-WC composite, because of high-conducting WC lying on the grain boundary, as seen in the current distribution plot (Figure 3d), the low resistance path is indicated via WC. The *I–V* characteristic for single-phase Ge_0.87_Mn_0.05_Sb_0.08_Te shows a typical semiconductor behavior (top inset, Figure 4c), which agrees with their electrical conductivity behavior in the literature. However, the composite with WC as a second phase located at the grain boundaries of Ge_0.87_Mn_0.05_Sb_0.08_Te shows metallic behavior at the grain boundary due to linear *I–V* dependence. This can be explained by the ohmic contact between GeTe and WC (which agrees with the results from the Kelvin probe as well with the obtained band structure for these materials presented in the next section) in combination with the presence of partial percolation paths of WC grains.

To further emphasize how significant is the difference in the mechanism of the current flow between a single-phase Ge_0.87_Mn_0.05_Sb_0.08_Te and between GeTe-WC composite, a semi-log plot is presented in Figure 4c. For a voltage range of ±20 mV (higher DC voltages were not applied to the composite as AFM is able to measure the current signal only in the limited range *ca*. −500 to 500 nA), measured current differs between materials by a few orders of magnitude, which shows how much easier the current flows through the polycrystalline Ge_0.87_Mn_0.05_Sb_0.08_Te when metallic WC is located at the grain boundaries. The above observations are consistent with the current distribution map shown in Figure 3d.

### 2.3 Electrical Transport Properties

**Figure 5:**
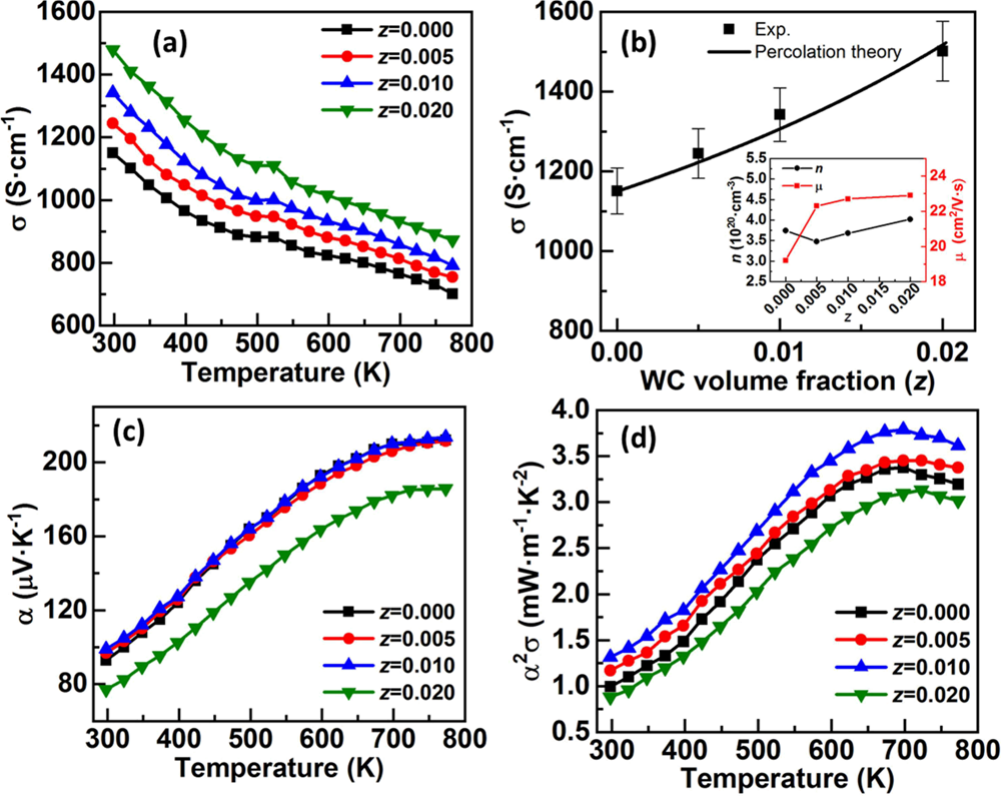
(a) Electrical conductivity (σ) as a function of temperature for the (1-*z*)Ge_0.87_Mn_0.05_Sb_0.08_Te-(*z*)WC composite. (b) σ as a function of WC volume fraction (*z*) at 300 K. σ calculated using the percolation model is shown using a solid line. Inset shows the changes in carrier concentration (*n*) and carrier mobility (μ) as a function of *z*. (c) Temperature-dependent Seebeck coefficient (α) and (d) power factor (α^2^σ) for the (1-*z*)Ge_0.87_Mn_0.05_Sb_0.08_Te-(*z*)WC composite.

The electrical conductivity (σ) for (1-*z*)Ge_0.87_Mn_0.05_Sb_0.08_Te-(*z*)WC (0 ≤ *z* ≤ 0.02) as a function of temperature is shown in Figure 5a. The σ for all the samples decreases with temperature, showing the degenerate semiconducting behavior of the materials. However, the σ enhances with an increase in the WC volume fraction in the composite. The σ of Ge_0.87_Mn_0.05_Sb_0.08_Te at 300 K is 1150 S·cm^–1^, and it increases to 1342 S·cm^–1^ for *z* = 0.010 and 1501 S·cm^–1^ for *z* = 0.020. This increase in σ may be attributed to the high σ of the dispersed phase (∼50,000 S·cm^–1^ for WC).^42^

The electrical conductivity (σ) as a function of WC volume fraction at 300 K is shown in Figure 5b. Because of the microstructure features of the composite, it is expected that the current will be flowing through the grain boundaries, where the amount of the highly conductive WC phase is relatively high. The theoretical values of the electrical conductivity of the composite are calculated using the percolation model,^43^ which is given by
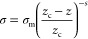
2

Here, σ_m_ and σ represent the electrical conductivity of the matrix and the composite, respectively, *z* is the volume fraction, and *s* is a constant, with a well-established and universal value of 0.87.^43^ This model was fitted to the experimental data using Origin software with percolation threshold as a parameter. The solid line in Figure 5b represents obtained values of electrical conductivity for the (1-*z*)Ge_0.87_Mn_0.05_Sb_0.08_Te-(*z*)WC composite using percolation theory with percolation threshold (*z*_c_) of 0.073 (Pearson’s correlation coefficient R^2^ = 0.971). This indicates that the volume fraction of WC required for the creation of continuous percolation paths in the composite is relatively small (∼7%). The volume fraction used in the present study (*z* = 0.005–0.02) is lower than the threshold value (∼7%), and hence, partial percolation is observed in the current distribution map, as shown in Figure 3d. However, this small volume fraction used in the present study is enough to cause a noticeable increase in composite electrical conductivity.

Next, we discuss the change in carrier concentration (*n*) and carrier mobility (μ) with an increase in WC volume fraction (*z*) in the composite using the relation σ = *ne*μ, where *e* is the electronic charge.^44^ The carrier concentration (*n*) measured using Hall measurement and corresponding carrier mobility (μ) estimated using the σ and *n* are shown in the inset of Figure 5b. It is found that the μ of the composite increases with an increase in the WC volume fraction. In general, the addition of the second phase creates scattering centers for charge carriers in bulk semiconducting materials and hence reduces μ. However, a significant enhancement in σ is obtained in the present study and is attributed to the enhanced μ. A similar observation was demonstrated by Zhou et al. in Ag-added skutterudites.^45^ The increase in μ in the composite sample may be attributed to the filtering of high-energy carriers at the interface between these two phases in the composite (discussed later).^46,47^

The temperature-dependent Seebeck coefficient (α) for (1-*z*)Ge_0.87_Mn_0.05_Sb_0.08_Te-(*z*)WC (0 ≤ *z* ≤ 0.02) is shown in Figure 5c. The α for all samples increases with an increase in temperature and is consistent with the changes in σ. Although the addition of WC improves the σ significantly, it also enhances the α of the composite sample up to *z* = 0.010. The α for Ge_0.87_Mn_0.05_Sb_0.08_Te at 300 K is ∼93 μV·K^–1^ and increases to (∼100 μV·K^–1^) up to *z* = 0.010. However, with a further increase in WC volume fraction, α decreases to 78 μV·K^–1^ for *z* = 0.020. These values of α are consistent with the α_av_ obtained from the scanning thermoelectric microprobe (STM) analysis (Supporting Information (SI), Figure S1). The increase and decrease in α are consistent with the carrier concentration change in the composite (Figure 5b). The carrier concentration decreases up to *z* = 0.010 and then increases. Such an increase in α for a small WC volume fraction can be attributed to the carrier energy filtering effect.^46,47^ Li et al. showed that the addition of SiC enhances α in the PbTe-based composite.^23^ A similar observation was reported in the Ge_0.94_Bi_0.06_Te-SiC composite.^27^ The simultaneous increase in σ and α results in an enhanced power factor (α^2^σ), as shown in Figure 5d. The α^2^σ increases with the increase in temperature up to ∼700 K and then decreases. The power factor increases from 1.32 mW·m^–1^·K^–2^ at 300 K and reaches ∼3.8 mW·m^–1^·K^–2^ at 700 K for the composite with *z* = 0.010. Because of a significant decrease in α for higher WC volume fraction (*z* = 0.020), α^2^σ decreases.

#### 2.3.1 Kelvin Probe Force Microscopy Measurement

It is noted that the increase in α^2^σ for the composite is owing to a significant rise in σ. Hence, a better understanding of how σ increases in the composite is required. For this purpose, we have investigated the potential barrier at the interface between the Ge_0.87_Mn_0.05_Sb_0.08_Te and WC by measuring the work function (φ) for both the individual phases using the Kelvin probe force microscopy (KPFM) technique.^48^ KPFM is a useful tool to estimate the relative position of the Fermi level in solids.^49^ The work function for individual phases is calculated by measuring the contact potential difference (CPD) using the KPFM. The CPD is defined as
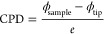
3where φ_tip_ and φ_sample_ are the AFM probe and sample work functions, respectively. The work function of the AFM probe (φ_tip_ = 4.15 eV) was calibrated by measuring the potential map of freshly cleaved and highly oriented pyrolytic graphite HOPG with the known value of work function (φ_HOPG_ = 4.6 eV).^50^ Furthermore, the work functions of the samples (φ_sample_) are obtained using φ_sample_ = φ_tip_ + CPD. The surfaces of both the samples (Ge_0.87_Mn_0.05_Sb_0.08_Te and WC) and HOPG were scanned alternatively to determine the CPD values.

**Figure 6:**
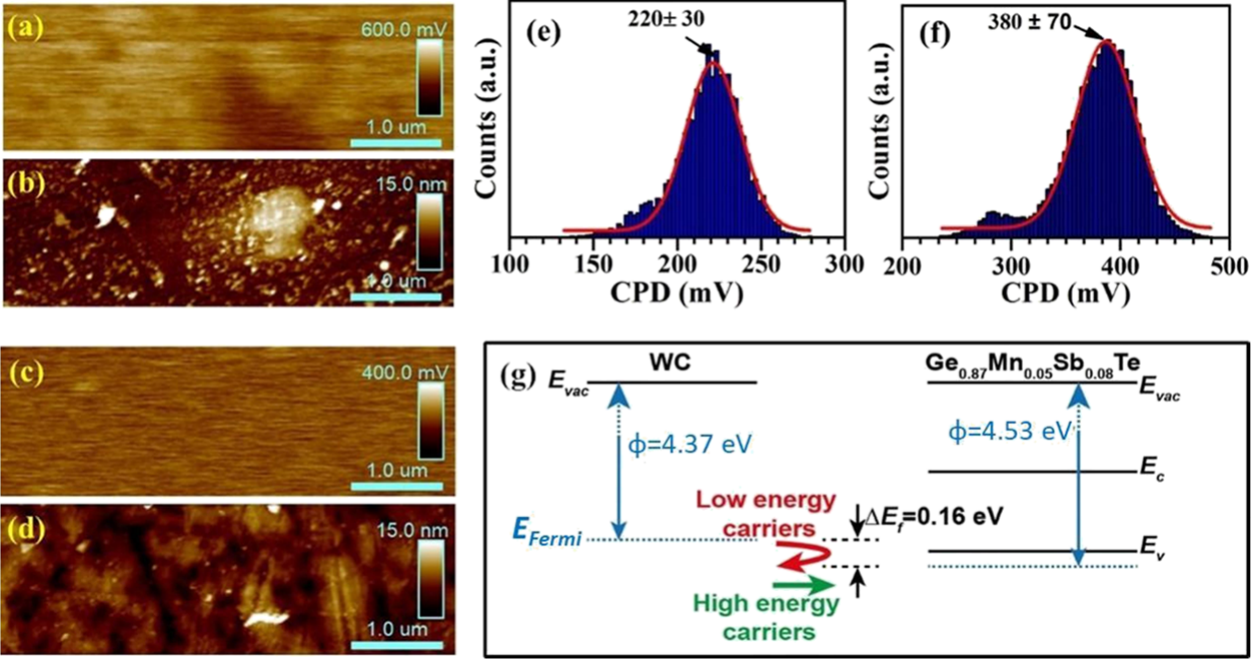
Spatial variation of contact potential difference (CPD) and corresponding two-dimensional surface topography for (a, b) Ge_0.87_Mn_0.05_Sb_0.08_Te and (c, d) WC. CPD histogram for (e) WC and (f) Ge_0.87_Mn_0.05_Sb_0.08_Te. (g) Band diagram was estimated from the work function obtained from the Kelvin probe force microscopy (KFPM) for Ge_0.87_Mn_0.05_Sb_0.08_Te and WC. Small Fermi energy difference indicates a cross-over of high-energy carriers at the interface.

The spatial variation of CPD and two-dimensional surface topography observed for both phases are shown in Figure 6a–d. The CPD histograms for Ge_0.87_Mn_0.05_Sb_0.08_Te and WC phases are shown in Figure 6e, f. The work function obtained from the KPFM measurement for Ge_0.87_Mn_0.05_Sb_0.08_Te and WC is 4.5 ± 0.14 and 4.37 ± 0.06 eV, respectively. It is worth noting that the mean values of CPD for HOPG analyzed before and after measurements are close (449 vs 461 mV). The work function obtained from the KPFM measurement is used to design the band diagram for both the phases and is shown in Figure 6g. It is noted that for the semiconductor (Ge_0.87_Mn_0.05_Sb_0.08_Te)–metal (WC) junction, there can be either Schottky contact (work function φ of the metal is greater than that of the semiconductor) or Ohmic contact (work function of the semiconductor is greater than that of metal).^51,52^ The present study shows that the work function for WC is smaller than that of Ge_0.87_Mn_0.05_Sb_0.08_Te, indicating an Ohmic contact and hence further supports the linear nature of the *I–V* curve in Figure 4c. It indicates that charge carriers can flow from WC to Ge_0.87_Mn_0.05_Sb_0.08_Te, supporting the enhanced σ in the system. Also, the energy difference (Δ*E*_f_) between these two materials is quite small (0.16 eV), which helps to enhance σ in the composites. This small difference in Δ*E*_f_ may scatter the lower energy carrier at the interface and allows the high energy carriers with increased μ to improve α due to energy filtering.^26,53^ This indicates that the slight mismatch in Δ*E*_f_ enhances both σ and α in the composite.

#### 2.3.2 Electronic Structure and Work Function Calculations

**Figure 7:**
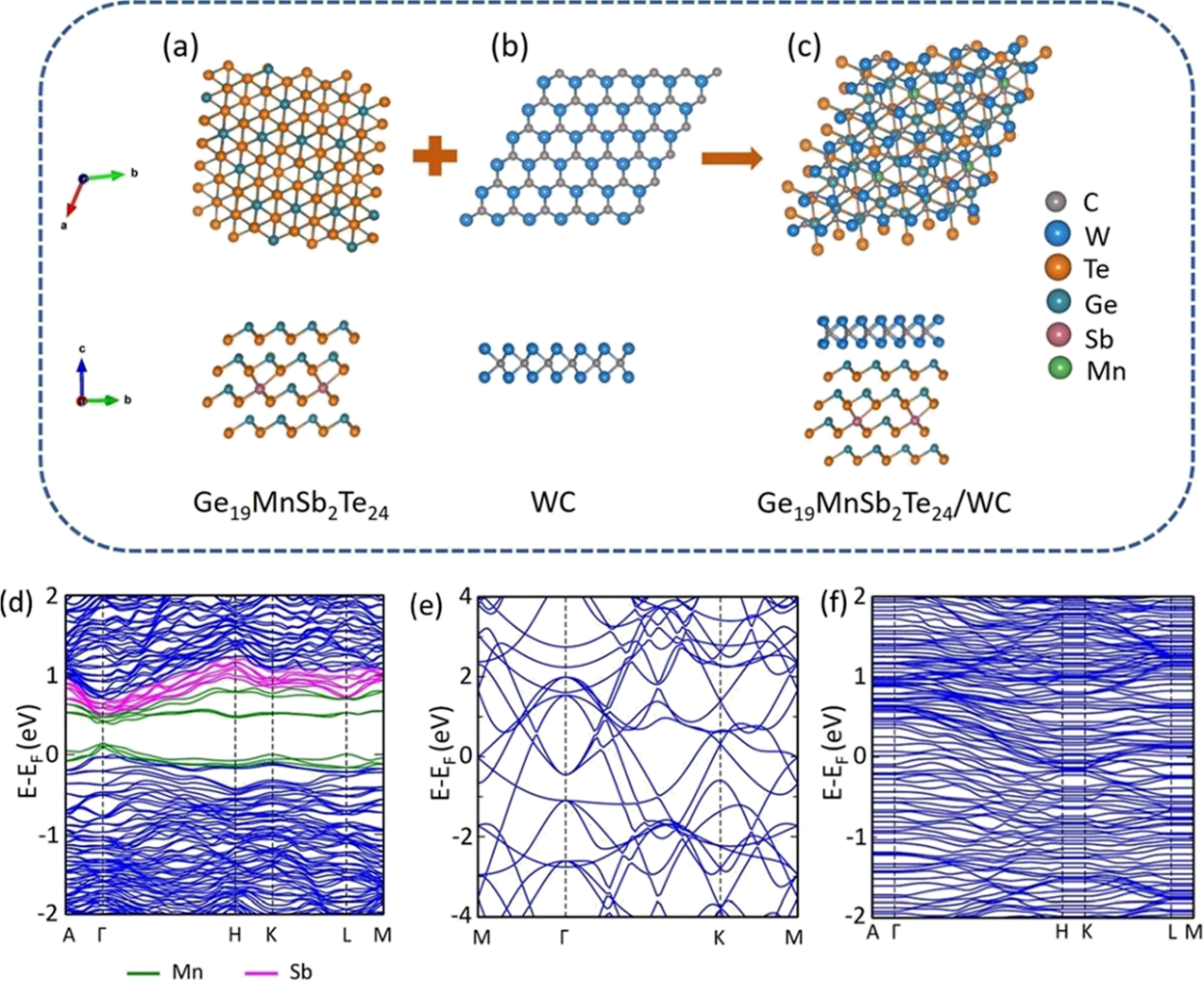
Side and top views of optimized geometries of (a) Ge_19_MnSb_2_Te_24_, (b) WC, and (c) Ge_19_MnSb_2_Te_24_-WC composite. Electronic band structures of (d) Ge_19_MnSb_2_Te_24_, (e) WC and (f) Ge_19_MnSb_2_Te_24_-WC composite.

For further exploration of the enhancement of electrical conductivity in the Ge_0.87_Mn_0.05_Sb_0.08_Te-WC composite, density functional theory (DFT) calculations are performed. The details of electronic structure calculations are shown in the SI (Figures S2–S4). First, we have examined the stability of the Ge_19_MnSb_2_Te_24_-WC composite by calculating its binding energy (*E*_b_), which is defined as
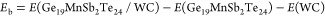
where *E*(Ge_19_MnSb_2_Te_24_-WC), *E*(Ge_19_MnSb_2_Te_24_) and *E*(WC) are, respectively, the total energies of the Ge_19_MnSb_2_Te_24_/WC composite, Ge_19_MnSb_2_Te_24_ matrix, and WC particles. Ge_19_MnSb_2_Te_24_ corresponds to Ge_0.87_Mn_0.05_Sb_0.08_Te, considering the intrinsic Ge vacancies during calculations, as shown in the earlier study.^41^Figure 7a–c shows the supercells of Ge_19_MnSb_2_Te_24_, WC, and layered Ge_19_MnSb_2_Te_24_-WC system, which represents an interface between these materials in the composite.

The obtained value of the binding energy for the Ge_19_MnSb_2_Te_24_-WC composite is *E*_b_ = −1.85 eV. The negative value of *E*_b_ implies that the composite is thermodynamically stable. Subsequently, we have calculated the band structures for Ge_19_MnSb_2_Te_24_, WC, and Ge_19_MnSb_2_Te_24_-WC supercells (Figure 7d–f). As we can see from Figure 7e, there is no gap in the band structure of WC, which indicates its metallic nature. On the other hand, Ge_19_MnSb_2_Te_24_ is a *p*-type semiconductor.^41^ This leads to the possibility of the charge transfer from WC particles to the Ge_19_MnSb_2_Te_24_ matrix in the Ge_19_MnSb_2_Te_24_-WC composite. Figure 7f shows that the obtained electronic structure for the layered supercell has no band gap, suggesting that the Ge_19_MnSb_2_Te_24_-WC composite should show metallic (degenerate semiconductor) behavior. The result agrees well with the linear *I–V* characteristic recorded for the composite, presented in the inset of Figure 4c.

**Figure 8:**
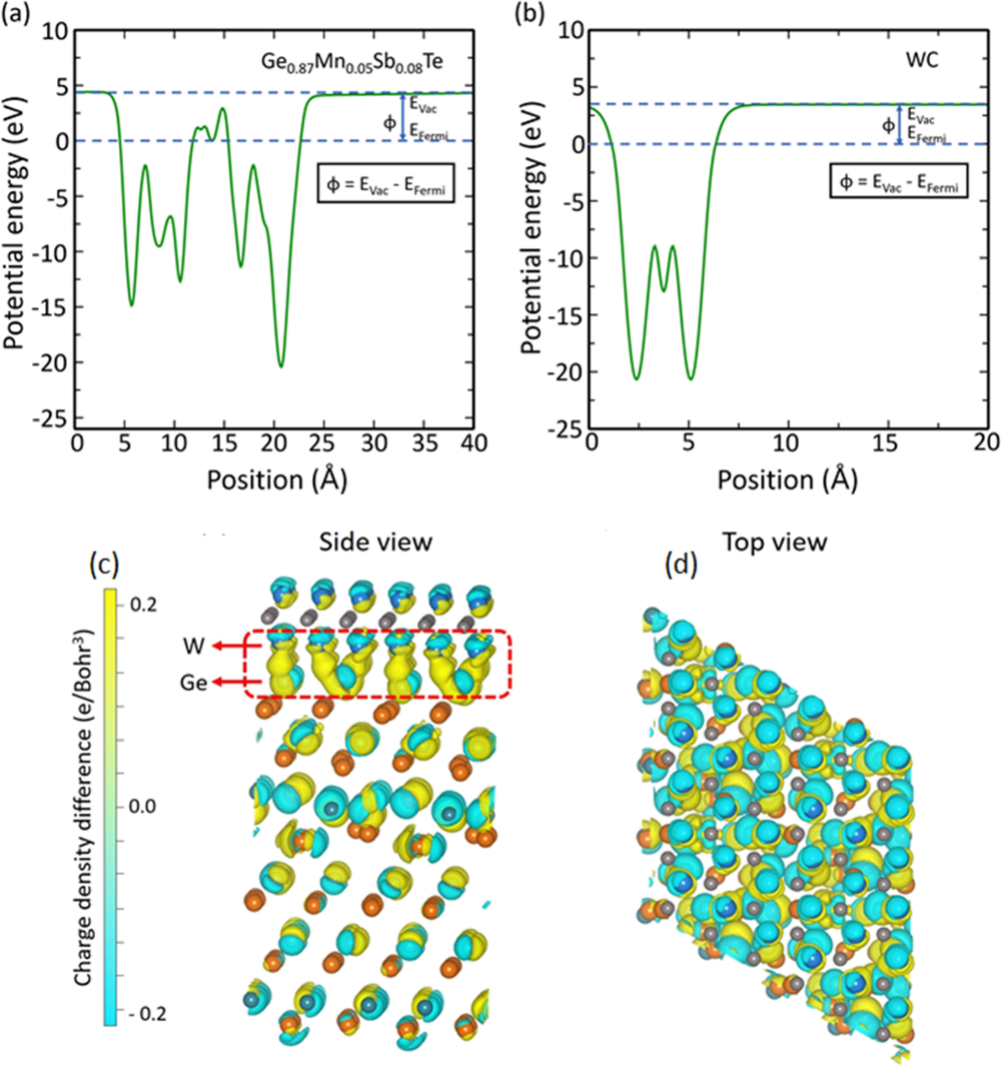
Electrostatic potential energy of (a) Ge_19_MnSb_2_Te_24_ and (b) WC along *Z*-direction. (c) Side and (d) top views of charge density difference plot for the Ge_19_MnSb_2_Te_24_-WC composite, where the cyan and yellow fields represent the electron accumulation and depletion, respectively.

To examine the charge transfer between WC and Ge_19_MnSb_2_Te_24_, we have plotted the electrostatic potential energy for both materials separately. Figure 8a, b shows the electrostatic potential energy of Ge_19_MnSb_2_Te_24_ and WC along the c-direction from Figure 6a, b. From these calculations, the obtained work functions for Ge_19_MnSb_2_Te_24_ and WC are 4.51 and 4.36 eV, respectively, which are in good agreement with the experimental results given using the KPFM method. The smaller work function φ of WC in relation to the Ge_19_MnSb_2_Te_24_ matrix indicates that charge can flow from WC inclusions to the matrix, leading to an increase in the effective electrical conductivity of the composite. To better visualize the charge transfer, we have plotted the 3D charge density difference at the interface between the Ge_19_MnSb_2_Te_24_ and WC, as shown in Figure 8c, d. The yellow and cyan fields represent the accumulation and depletion of electrons, respectively. In Figure 8c, it can be observed that charge transfer paths are created between W and Ge atoms (yellow areas). This phenomenon can be responsible for the enhancement of the electrical conductivity in the Ge_19_MnSb_2_Te_24_-WC composite.

### 2.4 Thermal Transport Properties

**Figure 9:**
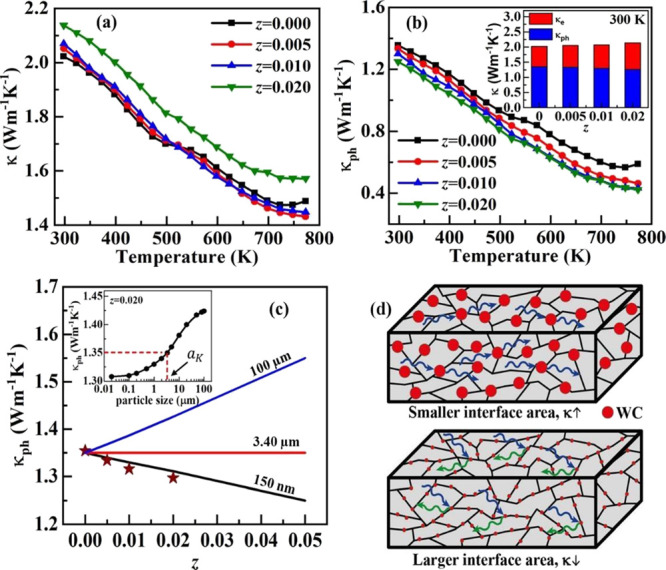
(a) Total thermal conductivity (κ), (b) phonon thermal conductivity (κ_ph_) as a function of temperature for the (1-*z*)Ge_0.87_Mn_0.05_Sb_0.08_Te-(*z*)WC composite. Inset of (b) shows the contribution of κ_e_ and κ_ph_ at 300 K, (c) κ_ph_ calculated (solid lines) as a function of WC volume fraction for (1-*z*)Ge_0.87_Mn_0.05_Sb_0.08_Te-(*z*)WC for the different particle size of WC, using Bruggeman’s asymmetrical model. The symbols represent the κ_ph_ obtained from the total κ. Inset of (c) shows the κ_ph_ calculated for composite with *z* = 0.020 as a function of the WC particle size. (d) Schematic of composite materials with different particle sizes of WC (red circles) is shown. Phonons are reflected for a smaller WC particle size because of the prominent effect of interface thermal resistance between phases due to the increased interfacial area/surface to volume ratio.

Temperature-dependent total thermal conductivity κ(*T*) for the (1-*z*)Ge_0.87_Mn_0.05_Sb_0.08_Te-(*z*)WC composite is shown in Figure 9a. κ decreases with temperature for all the samples; however, it is overall higher for a bigger volume fraction *z* of WC in the composite. A noticeable increase in κ in the whole temperature range is seen for the sample with *z* = 0.020. This increase in κ seems to be obvious because WC possesses extremely high κ (∼170 W·m^–1^·K^–1^ at 300 K).^42^ However, this sample does not follow the trend with materials with lower WC amounts. Because κ consists of two components, electronic thermal conductivity (κ_e_) and phonon thermal conductivity (κ_ph_), that is, κ = κ_e_ + κ_ph_, the addition of WC to the composite improves σ and hence must increase κ_e_. To clarify the nature of the electron and phonon contribution to thermal conductivity, both κ_e_ and κ_ph_ in the composite system are separated from κ by calculating the κ_e_ using Wiedemann Franz law κ_e_ = *L*σT, where the Lorenz number (*L*) was calculated using the equation proposed by Snyder and co-workers: *L* = × 10^–8^ W·Ω·K^–2^, where α is the Seebeck coefficient in μV·K^–1^.^54^

#### 2.4.1 Phonon Thermal Conductivity and Bruggeman’s Model

Temperature-dependent κ_ph_ for the (1-*z*)Ge_0.87_Mn_0.05_Sb_0.08_Te-(*z*)WC composite is shown in Figure 9b. It is worth noting that the κ_ph_ of the composite decreases with the increase in temperature, and interestingly, it is overall lower for a higher volume fraction of WC. The changes in electronic (κ_e_) and phonon thermal conductivity (κ_ph_) as a function of the WC volume fraction at 300 K are shown in the inset of Figure 9b. It is noted that κ_ph_ of WC is relatively high (∼135 W·m^–1^·K^–1^ at 300 K)^42^ compared to Ge_0.87_Mn_0.05_Sb_0.08_Te despite the fact that κ_ph_ of the composite decreases with the addition of the WC phase. This peculiar decrease in κ_ph_ is analyzed using Bruggeman’s asymmetrical model that considers the interface thermal resistance (*R*_int_) between the phases in the composite. The *R*_int_ for the composite is estimated using the acoustic impedance model (AIM) and the Debye model.^55,56^ The AIM shows that the phonon propagating from one material to another can be reflected if there is a mismatch in the acoustic impedance *Z* = *v*·ρ between the two materials.^29^ The sound velocity (*v*) and sample density (ρ) measured for both Ge_0.87_Mn_0.05_Sb_0.08_Te and WC and their calculated acoustic impedance are presented in Table 1. The probability of phonon transmission (η) at the interface between Ge_0.87_Mn_0.05_Sb_0.08_Te (matrix) and WC (dispersed phase) is η= 4.36%. It is given by the formula η = *pq*, where , which are calculated using the sound velocity of the matrix (*v*_m_) and dispersed phase (*v*_d_)^49^ and the acoustic impedance for the matrix *Z*_m_ and that of the dispersed phase *Z*_d_.

**Table 1:** Measured Values of Sample Density (ρ) and Sound Velocity (*v*) Used in the AIM Model To Calculate the Acoustic Impedance (*Z*) and Transmission Coefficient (*p*) of Phonons for Ge_0.87_Mn_0.05_Sb_0.08_Te and WC Samples

		*v* (m·s^–1^)		*Z* (kg·m^–2^ s^–1^)		
sample name	ρ (g·cm^–3^)	transverse (*v*_l_)	longitudinal (*v*_t_)	transverse	longitudinal	*p*_av_ (%)
Ge_0.87_Mn_0.05_Sb_0.08_Te	5.74	1870	3220	10,734	18,483	48.3
WC	15.43	4400	7180	67,892	110,787	

The probability of phonon transmission obtained from the AIM model is used to calculate the *R*_int_ between the Ge_0.87_Mn_0.05_Sb_0.08_Te-WC phases following the Debye model *R*_int_, where *c*_p_ is the specific heat capacity of the matrix and is the Debye velocity. Using the values of *c*_p_, ρ, *v*_D_, and η, the estimated *R*_int_ for Ge_0.87_Mn_0.05_Sb_0.08_Te and WC is 2.535·10^–6^ m^2^·K·W^–1^. This value of *R*_int_ is higher than that for several other TE composites and is beneficial for improving phonon scattering between the phases in the composite.^12^ The *R*_int_ also gives an important parameter of the critical grain size called Kapitza radius *a*_K_ calculated from the formula *a*_K_ = *R*_int_·κ_ph,m_. If highly conductive particles are introduced into the low-conducting matrix, the effective conductivity of the composite can be decreased if the size of the particles is lower than that of *a*_K_.^28^ Using the phonon thermal conductivity of the matrix (κ_ph,m_) and *R*_int_, *a*_K_ for Ge_0.87_Mn_0.05_Sb_0.08_Te-WC composite is 3.40 μm at 300 K. A high *R*_int_ provides a larger *a*_K_ and it suggests that κ_ph_ of the composite can be reduced if the particle size of WC is smaller than 3.40 μm. The consideration of AIM and the Debye model is important to estimate the critical size for a composite to reduce its κ_ph_.

Furthermore, Bruggeman’s asymmetrical model is used to analyze the κ_ph_ of the Ge_0.87_Mn_0.05_Sb_0.08_Te/WC composite, using *a*_K_ obtained for the composite, by the formula^30^
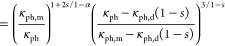
4where κ_ph,d_ and κ_ph_ are the thermal conductivity of the dispersed phase (WC) and the composite, respectively, and *s* = *a*/*a*_K_, where *a* is the actual particle size of the dispersed phase. κ_ph_ calculated using Bruggeman’s asymmetrical model for Ge_0.87_Mn_0.05_Sb_0.08_Te-WC composite with *z* = 0.020 at 300 K as a function of different particle sizes of WC is shown in the inset of Figure 9c. As can be seen from the curve, κ_ph_ decreases with the decrease in the particle size of WC. It reduces below κ_ph,m_ (1.35 W·m^–1^·K^–1^) when *a* < *a*_K_ (marked by the dotted line in the inset of Figure 9c).

Figure 9c depicts κ_ph_ as a function of WC volume fraction (*z*) for different particle sizes of WC at 300 K. The κ_ph_ increases when *a* > *a*_K_. It indicates that even a relatively high acoustic mismatch between Ge_0.87_Mn_0.05_Sb_0.08_Te and WC will cause an increase of κ_ph_ when the particle size of WC is too large. However, κ_ph_ remains constant for *a* ≈ *a*_K_, and it decreases when *a* < *a*_K_ and indicates that *R*_int_ becomes very prominent due to the very high contact area between the particles and the matrix and hence reduces the κ_ph_. It also suggests a strong correlation between *R*_int_ and *a*_K_.^12^ In other words, the smaller particle size of the dispersed phase enhances the surface-to-volume ratio and hence strongly reduces κ_ph_. A schematic of the Ge_0.87_Mn_0.05_Sb_0.08_Te-WC composite with different particle sizes of WC is shown in Figure 9d. It shows that the larger particle size of the dispersed phase possesses a lower interface area, which leads to an increase in κ_ph_ even for high *R*_int_. On the other hand, if the particle size of the dispersed phase is smaller than *a*_K_, the *R*_int_ becomes prominent with an increased interface surface area to the volume ratio and reduces κ_ph_.

#### 2.4.2 Phonon Dispersion Calculation

**Figure 10:**
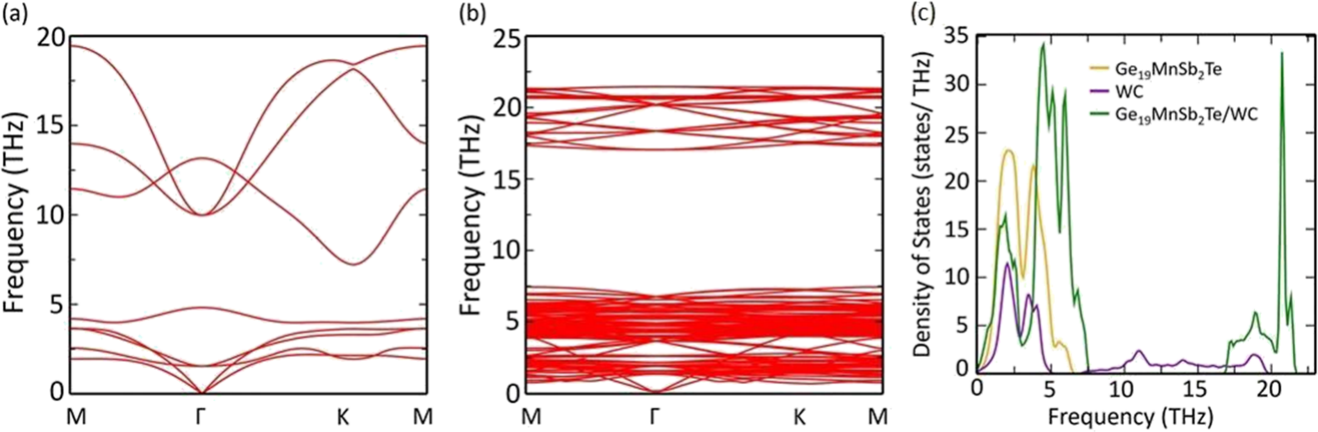
Phonon dispersion curves for (a) WC and (b) Ge_19_MnSb_2_Te_24_-WC composite. (c) Phonon density of states for Ge_19_MnSb_2_Te_24_, WC, and Ge_19_MnSb_2_Te_24_-WC composite.

Next, to examine the thermal stability, phonon dispersion curves for WC and Ge_19_MnSb_2_Te_24_-WC were plotted using the Phonopy code (see Figure 10a, b). Also, as shown in Figure 8c, the phonon density of states for Ge_19_MnSb_2_Te_24_, WC, and Ge_19_MnSb_2_Te_24_-WC composites are plotted, which shows their contribution in the different frequency ranges. In general, the phonon dispersion is indicated by the ω vs *k* plot, and the gradient of the ω vs *k* curve gives the *v*_g_ (phonon group velocity), where *v*_g_ = dω/d*k*. As can be seen in Figure 10a, b, the gradient of the phonon curve for Ge_19_MnSb_2_Te_24_/WC is lower than that of WC and Ge_19_MnSb_2_Te_24_.^41^ This suggests that the Ge_19_MnSb_2_Te_24_/WC has lower lattice thermal conductivity in comparison to Ge_19_MnSb_2_Te_24_ and WC. These findings are in good agreement with the experimental results.

### 2.5 Figure of Merit and Efficiency

**Figure 11:**
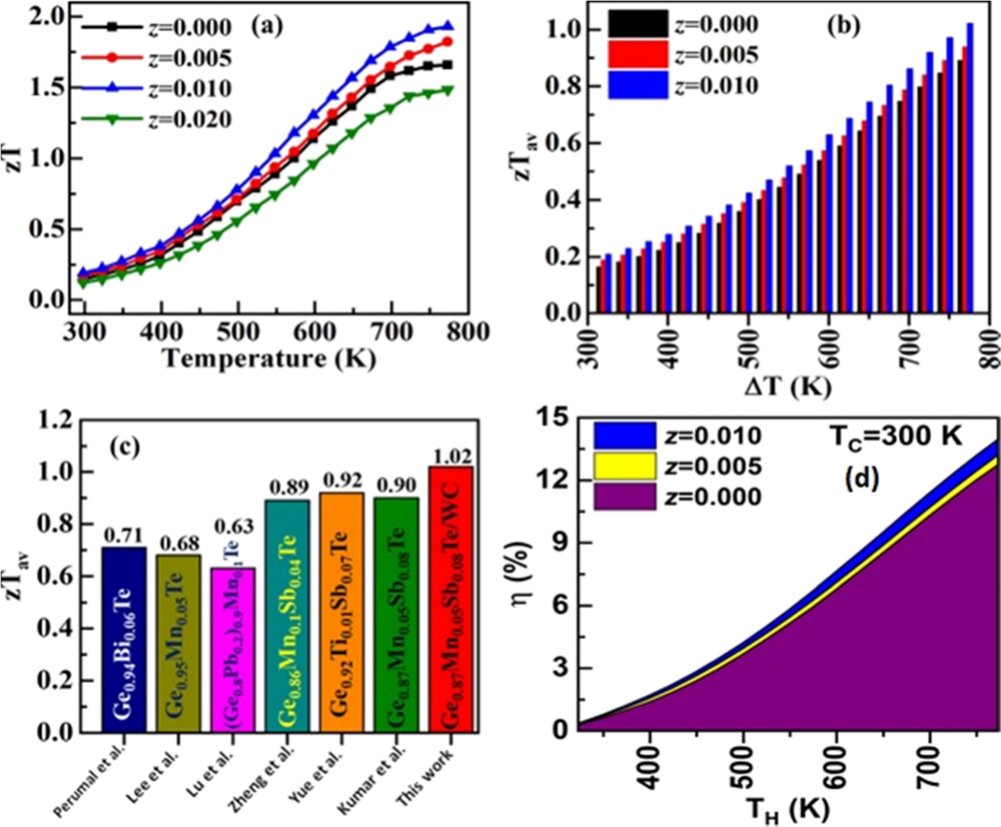
(a) Figure of merit (*zT*) (b) average *zT* for the (1-*z*)Ge_0.87_Mn_0.05_Sb_0.08_Te/(*z*)WC composite, (c) average *zT* (zT_av_) compared with the values reported in the literature.^57−61^ (d) Energy conversion efficiency calculated considering similar n-type leg for the (1-*z*)Ge_0.87_Mn_0.05_Sb_0.08_Te/(*z*)WC composite.

Using the experimentally observed TE parameters α, σ, and κ, the figure of merit (*zT*) of the composite is calculated and is shown in Figure 11a. The *zT* increases with temperature for all the samples. The *zT* also increases with WC volume fraction and shows a maximum of 1.93 at 773 K for the sample with *z* = 0.010. This enhancement in *zT* is attributed to the simultaneous rise in σ and α for composite along with reduced κ_ph_ owing to the AIM between the phases. However, the WC fraction higher than 0.010 reduces *zT* because of a significant reduction in α.

The energy conversion efficiency of TE devices made up of TE materials is defined as . This expression suggests that a high η requires a high *zT*_av_ () across a wide temperature difference (Δ*T* = *T*_H_ – *T*_C_) between the hot (*T*_H_) and cold (*T*_C_) side of the device. The *zT*_av_ calculated for the (1-*z*)Ge_0.87_Mn_0.05_Sb_0.08_Te-(*z*)WC composite with 300 and 773 K as *T*_C_ and cold *T*_H,_ respectively, is shown in Figure 11b. The *zT*_av_ for the sample with *z* = 0.010 is the highest. It reaches ∼1.02 for a temperature difference of Δ*T* = 473 K. The obtained *zT*_av_ in the present study is higher than that previously reported in several literature reports.^57−61^ A comparison for the same is shown in Figure 11c. It is noted that there are some recent article that shows higher zT_av_ in GeTe such as Sc-Bi co-doped GeTe,^36^ Cr-Bi co-doped GeTe (*zT*_av_ ∼ 1.2 from 300 to 723 K),^32^ Bi-Zn co-doped GeTe (*zT*_av_ = 1.35 from 400 to 800 K);^33^ however, because of differences in their temperature range studies, they are not included in Figure 11c. Furthermore, using the *zT*_av_ obtained in the present study for the p-type material and assuming a corresponding similar *n*-type leg, theoretical energy conversion efficiency (η) is calculated for the temperature difference (Δ*T*) and shown in Figure 11d. A maximum energy conversion efficiency of ∼14% is obtained for the (1-*z*)Ge_0.87_Mn_0.05_Sb_0.08_Te-(*z*)WC for *z* = 0.010 composite and is higher than those of several other promising TE materials reported in the literature.^57−61^

## 3 Conclusions

This study demonstrates the influence of work function and AIM on electronic and phonon transport properties, respectively, in the Ge_0.87_Mn_0.05_Sb_0.08_Te-WC composite. X-ray diffraction analysis confirms the individual phases in the composite, which is further supported by electron microscopy images and energy-dispersive X-ray spectroscopy analysis. Enhancement in the composite’s electrical conductivity (σ) is attributed to the increase in carrier mobility (μ). It is also analyzed using the work function measurement using the Kelvin probe force microscopy technique. The lower work function of WC compared to Ge_0.87_Mn_0.05_Sb_0.08_Te gives high mobility charge carriers to the system and hence increases σ. Additional AFM analysis showed higher current flow near the boundary of Ge_0.87_Mn_0.05_Sb_0.08_Te grains for composites in comparison to the single-phase material. The current–voltage (*I*–*V*) characteristics indicate the Ohmic contact between Ge_0.87_Mn_0.05_Sb_0.08_Te and WC grains. The density functional theory (DFT) calculations further support the increase in electrical conductivity and linear *I*–*V* characteristics in the composite. The acoustic impedance mismatch (AIM) between the composite phases leads to a high interface thermal resistance (*R*_int_), beneficial for improving phonon scattering. A correlation between *R*_int_ and the Kapitza radius (*a*_K_) decreases the phonon thermal conductivity (κ_ph_) of the composite, supported by Bruggeman’s asymmetrical model. Furthermore, the phonon dispersion calculations suggest a decrease in phonon group velocity in the composite. The simultaneous effect of the work function and AIM shows an improved power factor and reduced κ_ph_. As a result, a maximum *zT* of 1.93 at 773 K with a *zT*_av_ ∼ 1.02 for a temperature difference of 473 K is obtained for (1-*z*)Ge_0.87_Mn_0.05_Sb_0.08_Te-(*z*)WC with *z* = 0.010. A maximum energy conversion efficiency (η) of ∼14% is calculated, considering a similar n-type material. This study shows promise to further develop efficient thermoelectric composite over a wide range of materials having similar electronic structures and different elastic properties (considering the correlation between *R*_int_ and *a*_K_).

## 4 Experimental Section

### 4.1 Synthesis and Structural Characterization

The synthesis of Ge_0.87_Mn_0.05_Sb_0.08_Te has been carried out by the direct melting of elements (Ge, Mn, Sb, and Te) with purity >99.99% (Alfa Aesar) in evacuated quartz ampoules following our previous report in GeTe.^41^ Furthermore, the (1-*z*)Ge_0.87_Mn_0.05_Sb_0.08_Te-(*z*)WC composite with *z* = 0.000, 0.005, 0.010, and 0.020 is prepared by mixing the different volume percentage of WC (Sigma Aldrich, 99.9%). The composite mixture was ground to mix homogeneously in a liquid medium (acetone) using a mortar and a pestle. The mixture was then sintered using the pulsed electric current sintering (PECS) technique in the Ar (5 N) atmosphere at 873 K for 5 min under a uniaxial pressure of 50 MPa with a heating and a cooling rate of 70 and 50 K/min, respectively. The obtained cylindrical pellets of 10 mm diameter and 12 mm length were cut to proper dimensions using a precise wire saw for further measurements. The surface morphology and chemical analysis of the polished sample surface were done using a scanning electron microscope (NOVA NANO 200, FEI EUROPE Company) equipped with an EDXS analyzer. The X-ray diffraction of samples was obtained by the D8 ADVANCE (BRUKER) diffractometer using Ni-filtered Cu-K_α_ radiation (λ = 1.5406 Å). The bulk density of the sintered pellets was measured using sample mass and their geometrical volume.

### 4.2 Electrical and Thermal Transport Properties

The spatial variation of the Seebeck coefficient for the (1-*z*)Ge_0.87_Mn_0.05_Sb_0.08_Te-(*z*)WC composite was done using a scanning thermoelectric microprobe at 300 K with a spatial resolution of 50 μm. Thermal diffusivity for all the samples was measured using the laser flash analysis (LFA-457, NETZSCH) apparatus in the Ar (5 N) atmosphere (30 mL/min). Specific heat was determined simultaneously with the thermal diffusivity using pyroceram 9606 as a reference material. The sample density was measured using sample mass and its geometric volume. Electrical conductivity and Seebeck coefficients were measured under the Ar (5 N) atmosphere (50 mL/min) using the SBA 458 (NETZSCH) apparatus. The uncertainty of the Seebeck coefficient and electrical conductivity measurements is 7 and 5%, respectively. The estimated uncertainty in thermal conductivity is 7%. The carrier concentration was measured at 300 K using a physical properties measurement system (PPMS, Quantum Design) under the magnetic field of ±3 T. Work function measurements were performed using an atomic force microscope (Dimension ICON, Bruker) working in the Peak Force KPFM mode. PFQNE-AL probes (with a nominal spring constant of 0.8 N/m) were used for capturing topography and potential maps under ambient conditions. The images were captured with a resolution of 84 × 256 pixels. The work function of the AFM tip (ϕ_tip_ = 4.15 eV) was determined by measuring the CPD of freshly cleaved HOPG with the known value of the work function (4.6 eV). The CPDs of HOPG before and after measurements were virtually the same (0.45 and 0.46 V), excluding tip contamination. The work function of the analyzed sample was calculated using eq 3. AFM conductivity measurements were performed using the same machine as in the case of KPFM analysis. AFM was operating in the PeakForce Tuna mode. The electrical contact between the bottom of the sample and the AFM table was made by gluing the sample onto a metal disc using a silver paste. The measurements were performed in air using HQ: NSC36/Cr-Au probes (MikroMasch, nominal spring constant 0.6 N/m) under constant load adjusted to achieve stable tip-sample electrical contact. The DC voltage applied during capturing current maps was set to 20 mV. The out-of-plane measurements of *I*–*V* curves were performed using the same AFM probes.

### 4.3 Theoretical Methods

The DFT^62,63^ calculations were performed using the plane-wave-based pseudopotential approach, as implemented in the Vienna Ab initio Simulation Package (VASP).^64,65^ The self-consistency loop was converged with a total energy threshold of 0.01 meV. The structures were fully relaxed until the Heymann–Feynman forces on each atom were less than 10^–5^ eV/Å. The structural optimization was carried out using generalized gradient approximation (GGA) expressed by the Perdew–Burke–Ernzerhof (PBE)^66^ exchange-correlation functional. The effects of doping were considered by substituting Mn and Sb atoms at the specific sites of Ge atoms. 2 × 2 × 1 and 3 × 3 × 1 supercells were employed for GeTe and WC so that there exists a minimum lattice mismatch of ∼3.25%. A 6 × 6 × 1 *k*-mesh was used for Brillouin zone sampling for the Ge_0.87_Mn_0.05_Sb_0.08_Te-WC composite. The periodic units were separated by a vacuum layer with 20 Å thickness along the *Z*-direction to prevent spurious interactions between periodic images. The two-body vdW interaction as devised by Tkatchenko-Scheffler has been employed.^67,68^ The correction parameter is based on the Hirshfield partitioning of the electron density. The electron wave function was expanded in a plane-wave basis set with an energy cutoff of 600 eV. Spin–orbit coupling interactions owing to heavy atoms were included when calculating the electronic structures. Phonon calculations were obtained within the harmonic approximation and using a finite displacement method.^69^ A 2 × 2 × 2 supercell was set for the calculations. The starting parameters for the calculations were the values obtained from the refinement.

## Supporting Information Available

Spatial distribution of the Seebeck coefficient using a STM and details of electronic band structure calculations for the GeTe-WC composite (PDF)
