# Supplementary material for: Synergistic Effect of Work Function and Acoustic Impedance Mismatch for Improved Thermoelectric Performance in GeTe-WC Composite
Source: ACS Appl Mater Interfaces. 2022 Sep 21;14(39):44527–38. doi: 10.1021/acsami.2c11369 (PMC9542701; doi:10.1021/acsami.2c11369)
Supplement: Supplementary file 1 — am2c11369_si_001.pdf [file am2c11369_si_001.pdf]

Supporting Information

Synergistic effect of work function and acoustic impedance mismatch for improved thermoelectric performance in GeTe-WC composite

Ashutosh Kumar[a], Preeti Bhumla[c], Artur Kosonowski[b], Karol Wolski[d], Szczepan Zapotoczny[d], Saswata Bhattacharya[c*], and Krzysztof T. Wojciechowski[b*]

[a]Lukasiewicz Research Network- Krakow Institute of Technology, Krakow Poland
[b]Faculty of Materials Science and Ceramics, AGH University of Science and Technology, Kraków 30-059, Poland
[c]Department of Physics, Indian Institute of Technology Delhi, New Delhi 110016, India
[d]Faculty of Chemistry, Jagiellonian University, Gronostajowa 2, Krakow 30-387, Poland

*corresponding author(s):
Krzysztof T. Wojciechowski, e-mail: wojciech@agh.edu.pl
Saswata Bhattacharya, e-mail: saswata@physics.iitd.ac.in

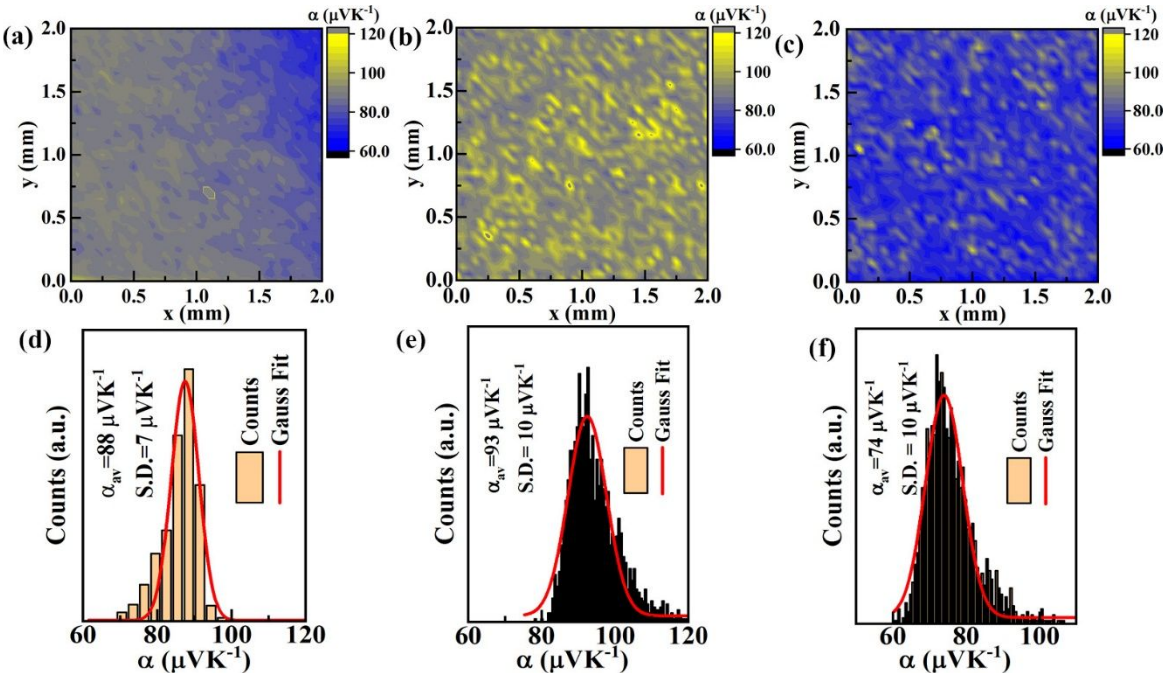

Fig. S1: Spatial distribution of the Seebeck coefficient and corresponding histogram curves for (1-z)$Ge_{0.87}Mn_{0.05}Sb_{0.08}Te$/(z)WC composite for (a,d) z=0.000, (b,e) z=0.010, (c,f) z=0.020.

Electronic structure calculations:

Choice of functional:

We have calculated the work functions for $Ge_{19}Mn_1Sb_2Te_{24}$ and WC using PBE exchange correlation (ε_{xc}) functional[1] as it reproduces the work function (with error bars ~ 0.2 eV) close to experimental ones. The obtained values of work functions for $Ge_{19}Mn_1Sb_2Te_{24}$ and WC are 4.51 eV and 4.36 eV, which matches well with the experimental work functions.

Crystallographic orientations:

In order to check the variation of work function with crystallographic orientations, we have considered different planes viz., 001, 010 and 100 for $Ge_{19}Mn_1Sb_2Te_{24}$. Fig S2. shows the different crystallographic orientations in $Ge_{19}Mn_1Sb_2Te_{24}$.

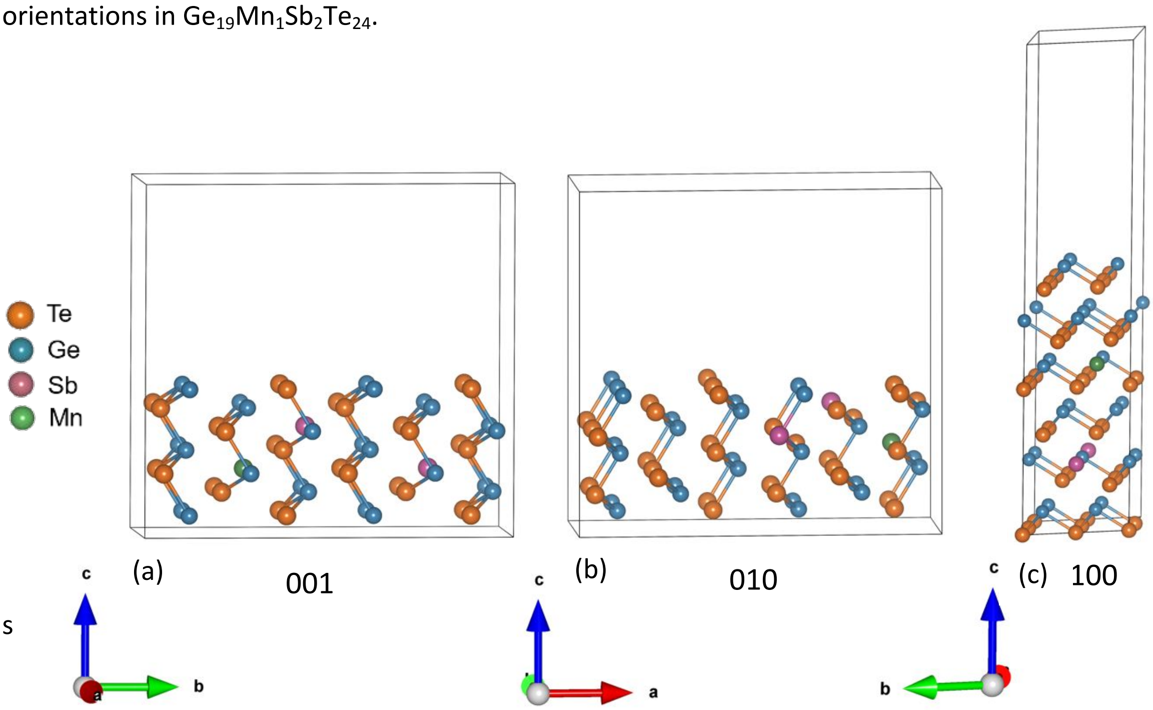

Fig S2. Different crystallographic orientations of $Ge_{19}Mn_1Sb_2Te_{24}$ (a) 001 (b) 010 (c) 100.

After modelling supercells along these planes, we have optimized the obtained structures with PBE ε_{xc} functional including van der Waals (vdW) interactions. The calculated energies for 001, 010 and 100 structures are -221.7445, -221.7427 and -230.3457 eV, respectively. The 100 plane is the most stable one having minimum energy. Also, we have checked work functions for all the configurations. The work functions obtained for 001, 010 and 100 structures are 4.49, 4.48 and 4.51 eV. We have proceeded with 100 configurations as it has work function closer to experimental value and possesses minimum energy.

Supercell convergence:

We have taken 2x2x1 and 2x2x2 supercells containing 24 and 48 atoms, respectively, for GeTe. After that, we substituted Mn and Sb at suitable sites and performed optimization in the obtained $Ge_{19}Mn_1Sb_2Te_{24}$ supercells. Fig S3 shows the 2x2x1 and 2x2x2 supercells of $Ge_{19}Mn_1Sb_2Te_{24}$. For defect localization, we have chosen a 2x2x2 supercell.

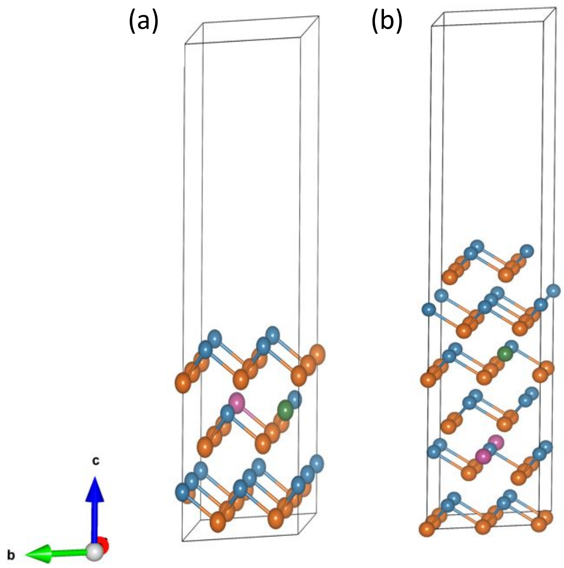

Fig S3. Supercells for $Ge_{19}Mn_1Sb_2Te_{24}$ (a) 2x2x1 (b) 2x2x2 containing 24 and 48 atoms, respectively.

Heterostructure:

To study the interaction between WC and $Ge_{19}Mn_1Sb_2Te_{24}$, we have modelled GeTeMnSb-WC heterostructure. For this, we have taken a 2x2x2 supercell containing 75 atoms. During optimization, the distance between WC and GeTeMnSb is automatically adjusted to get the minimum energy configuration of the composite. In our case, the distance between WC and GeTeMnSb is ~3Å. This obtained geometry is then used for further calculations. Fig S4. shows the optimized structure of the heterostructure.

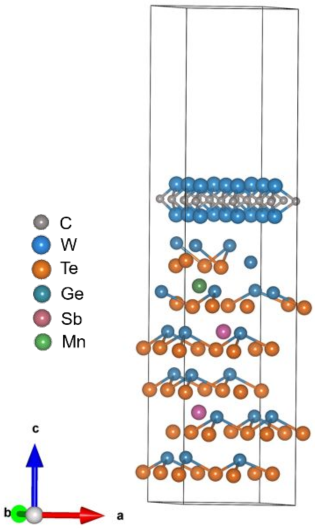

Fig S4. Supercell (2x2x2) of GeTeMnSb-WC containing 75 atoms.

References:

1. De Waele, S., Lejaeghere, K., Sluydts, M. & Cottenier, S. Error estimates for density-functional theory predictions of surface energy and work function. *Physical Review B* **94**, 235418 (2016).
